# Electrospun Bilayer Chitosan/Hyaluronan Material and Its Compatibility with Mesenchymal Stem Cells

**DOI:** 10.3390/ma12122016

**Published:** 2019-06-24

**Authors:** Valentina A. Petrova, Daniil D. Chernyakov, Daria N. Poshina, Iosif V. Gofman, Dmitry P. Romanov, Alexander I. Mishanin, Alexey S. Golovkin, Yury A. Skorik

**Affiliations:** 1 Institute of Macromolecular Compounds of the Russian Academy of Sciences, Bolshoy pr. V.O. 31, 199004 St Petersburg, Russia; valentina_petrova_49@mail.ru (V.A.P.); daniel.chernyakov@gmail.com (D.D.C.); poschin@yandex.ru (D.N.P.); gofman@imc.macro.ru (I.V.G.); 2 Institute of Silicate Chemistry of the Russian Academy of Sciences, Adm. Makarova emb. 2, 199034 St. Petersburg, Russia; dprom@mail.ru; 3 Almazov National Medical Research Centre, Akkuratova str. 2., 197341 St. Petersburg, Russia; mishaninssma@yandex.ru (A.I.M.); golovkin_a@mail.ru (A.S.G.)

**Keywords:** chitosan, hyaluronic acid, electrospinning, tissue engineering, polyelectrolyte complex, mesenchymal stem cells

## Abstract

A bilayer nonwoven material for tissue regeneration was prepared from chitosan (CS) and hyaluronic acid (HA) by needleless electrospinning wherein 10–15 wt% (with respect to polysaccharide) polyethylene oxide was added as spinning starter. A fiber morphology study confirmed the material’s uniform defect-free structure. The roughness of the bilayer material was in the range of 1.5–3 μm, which is favorable for cell growth. Electrospinning resulted in the higher orientation of the polymer structure compared with that of corresponding films, and this finding may be related to the orientation of the polymer chains during the spinning process. These structural changes increased the intermolecular interactions. Thus, despite a high swelling degree of 1.4–2.8 g/g, the bilayer matrix maintained its shape due to the large quantity of polyelectrolyte contacts between the chains of oppositely charged polymers. The porosity of the bilayer CS–HA nonwoven material was twice lower, while the Young’s modulus and break stress were twice higher than that of a CS monolayer scaffold. Therefore, during the electrospinning of the second layer, HA may have penetrated into the pores of the CS layer, thereby increasing the polyelectrolyte contacts between the two polymers. The bilayer CS–HA scaffold exhibited good compatibility with mesenchymal stem cells. This characteristic makes the developed material promising for tissue engineering applications.

## 1. Introduction

The development of scaffolds based on biocompatible materials for use in cell proliferation in artificial tissue, wound healing, and effective implantation is an essential task in tissue regeneration therapy. Cells grown on biocompatible and biodegradable polymer scaffolds (i.e., matrices that degrade in the organism without the formation of toxic products) retain good viability, as such scaffold materials prevent cell rupture during transplantation and accelerate cell growth in the new environment [1]. 

One rapidly developing field in the design of tissue engineering constructs is stem cell technology. Stem cells are capable of both long-term self-renewal and multilineage differentiation by asymmetric division [2]. Multipotent adult stem cells (i.e., mesenchymal stem cells (MSCs)) are a vital component of current technologies aimed at wound healing and tissue repair. MSCs have multipotent, trophic, and immunomodulatory properties, genomic integrity and stability, which make them promising for use in regenerative medicine [3]. However, after several passages in in vitro culture, stem cells tend to lose their ability to differentiate and self-renew [4]. This ability is controlled by the microenvironment surrounding the MSCs; thus, the difficulty in fully recreating and controlling the native cell microenvironment to control the ultimate stem cell fate is a critical challenge in MSC therapy [5,6]. 

Mimicking the properties of the MSC microenvironment using natural or synthetic materials as cell scaffolds for tissue engineering is difficult, as these properties are determined mainly by the extracellular matrix (ECM). The ECM is a natural fiber net that has a diameter of 50–500 nm, consists primarily of collagen and elastin, and contains some heparin, chondroitin sulfate, and HA. This matrix provides structural support for tissues, promotes efficient cell adhesion, and facilitates cellular interactions [7]. Therefore, artificial scaffold materials must also perform these functions. Proposed materials that can mimic the ECM and promote cell growth include polypeptides, hydroxyapatites, alginates, glucosaminoglycans, fibronectin, collagen, chitosan (CS), and hyaluronic acid (HA). CS and HA are broadly applied due to their unique properties. 

CS, which is a linear polysaccharide composed of β-linked D-glucosamine and N-acetyl-D-glucosamine, shows high potential for tissue regeneration. As a biomaterial, it can be introduced in different forms, such as film, fiber, and gel, and it is degraded by lysozyme to N-acetylglucosamine and glucosamine, which are naturally occurring metabolites in most organisms [8,9,10,11]. However, CS is rapidly dissolved in human tissues, especially in the acidic medium that usually forms during wound healing [12]. Nevertheless, the CS lifetime in wound tissue can be prolonged, and CS structural stability can be increased using different crosslinking agents and a combination of other polymers, such as collagen, gelatin, and glucosaminoglycans [13].

HA, a linear glycosaminoglycan consisting of repeating units of N-acetyl-D-glucosamine and D-glucuronic acid, has unique physicochemical and biological properties, including biocompatibility, biodegradability, mucoadhesivity, hygroscopicity, and viscoelasticity. It is widespread in humans and vertebrates, and participates in natural regeneration processes, cell differentiation, morphogenesis, and inflammation. In medicine, HA is used for the prevention of postoperative adhesions and as wound dressing, synovial fluid substitute, eye surgery medium, and as a medium for the preservation and transportation of cell lines [14]. HA has been shown recently to act both as a passive structural molecule and as a signal molecule, with its function depending on its molecular weight (MW) [15]. Its pronounced hygroscopicity and viscoelasticity allow HA to control tissue hydration, osmotic balance, and the physical properties of the ECM by structuring and stabilizing the extracellular space [16,17,18]. By its interactions with proteins and its influences on specific cell factors, HA functions as a signal molecule and participates in receptor expression, signal pathways, and the entire cell cycle. The binding of HA to proteins initiates different and opposite phenomena by inducing or reducing inflammation processes, stimulating or inhibiting cell migration, and activating or blocking cell division and differentiation [15].

The composition of an artificial scaffold primarily influences its biocompatibility. However, the morphology and structure of the scaffold also play a role [19]. Scaffolds for cell proliferation can be fabricated as different 3D constructs, including gels, micro- and nano-spheres, fibers, and films, and these structures have the combined properties of the polymers used in their production [20]. One method for generating 3D structures is electrospinning, an efficient and cost-effective technique of nanofiber production from natural and synthetic polymers [21]. The resulting electrospun fibers have large surface areas and high porosity and can mimic the microarchitecture of a native ECM [22]. This structural homology leads to improved adhesion, proliferation, migration, and differentiation of cells grown on electrospun matrices [23,24]. Recent reviews have focused on the parameters of initiation and stabilization of electrospinning using solutions of natural polymers, such as CS, collagen, alginate, and gelatin [25]. 

CS is one of the best-studied polymers in terms of electrospinning and has been electrospun from different solvents, including trifluoroacetic acid/dichloroethane [26] and 90% acetic acid [27]. The addition of so-called guest polymers, such as polyvinyl alcohol (PVA) [26] and polyethylene oxide (PEO) [28,29,30], stabilizes the electrospinning of CS and results in the formation of defect-free fibers. The matrix architecture can be controlled by the addition of nanofillers [31,32] or by crosslinking; these steps improve the mechanical properties and increase the time required for biodegradation [33,34]. Other approaches include the use of interpolymer complexes of non-ionogenic soluble polymers [35] or the combination of different polymers in the spinning solution [33,34]. 

The ability of CS to form polyelectrolyte complexes (PECs) is widely exploited in materials used in biomedicine [36,37]. We previously used a combination of structural and chemical modifications to produce bilayer composite films from CS and HA. The properties and structures of these films are described in our previous papers [38,39]. The production of bilayer composites involves forming a PEC through the electrostatic interaction between the layers of a polycation and a polyanion. The PEC initiates changes in the structure of both polymeric layers. Efficient scaffolds can also be designed using bilayer nonwoven materials, which combine the properties of the chemically different polymers used in their fabrication. Such materials have been proposed mostly for skin and cartilage regeneration [40], as they are stronger and have a lower solubility compared with monolayer nonwovens. The properties of the innermost and uppermost layers may also differ. 

The present work aimed to prepare and characterize an electrospun bilayer nonwoven material fabricated from CS and HA and to evaluate its compatibility with MSCs to determine its potential for use in tissue regeneration. In the case of CS and HA polyelectrolytes, the formation of a PEC layer can stabilize the material and reduce its solubility without the need for crosslinking agents while allowing changes in the morphology and architecture of the composite. Nonwoven bilayer materials are characterized by the formation of a rare mesh of polyelectrolyte contacts that reinforce the composite.

## 2. Materials and Methods

### 2.1. Characterization of Polysaccharides

The crab chitosan (Qingdao Honghai bio-tech Co., LTD, Qingdao City, China) had a viscosity average MW of 1.1 × 10^5^ and 98% degree of deacetylation (determined by ^1^H NMR spectroscopy, Bruker Avance II 400 MHz spectrometer, Bruker, Billerica, MA, USA). Sodium hyaluronate (Shandong Focuschem Biotech Co. Ltd., Qufu City, China) had a viscosity average MW of 5.4 × 10^4^.

The intrinsic viscosity of CS was determined by viscometry using an Ubbelohde capillary viscometer (Design Bureau Pushchino, Pushchino, Russia) at 20 °C with 0.33 M acetic acid/0.3 M NaCl as solvent. The MW of CS was calculated using the Mark–Houwink equation [η] = 3.41 × 10^−3^ × МW^1.02^ [41]; [η] = 4.7 dL/g. 

The intrinsic viscosity of HA was determined using the Ubbelohde viscometer (Design Bureau Pushchino, Pushchino, Russia) at 30 °C in a 0.2 M NaCl solution. The MW of HA was calculated from the equation [η] = 3.9 × 10^−2^ × MW^0.77^ [42]; [η] = 1.7 dL/g.

### 2.2. Polymer Solutions

The concentrations of the polymer solutions for electrospinning were experimentally selected to ensure uniform spinning; the concentrations of CS and HA were 2% and 4%, respectively. CS was first suspended in water by vigorous stirring for several hours, and then, the acetic acid was added under continuous stirring. Upon complete dissolution of the CS, a 3% PEO solution in water was added. The final component concentrations were as follows: a 2% solution of CS in 30% acetic acid with the addition of 10 relative wt% PEO (with respect to CS). HA was dissolved in water, and PEO was added under constant stirring to produce a 4% aqueous solution of HA with the addition of 15 relative wt% PEO (with respect to HA). Solutions of the starting polymers were purified by filtration under vacuum. The resulting solutions were used for the preparation of nonwovens and films.

### 2.3. Electrospinning

Electrospinning was performed on a Nanospider NS Lab 500 (Elmarco, Liberec, Czech Republic) using the non-capillary method and a 22–24 cm distance between the electrodes. The rotation speed of the spinning electrode was varied from 10 to 16 min^−1^, and the electrospinning voltage was 60–75 kV. Fibers were collected on a paper substrate. Three nonwoven materials were obtained by electrospinning: a CS–PEO monolayer matrix, a HA–PEO monolayer matrix, and a CS–HA–PEO bilayer matrix. The CS–HA-PEO bilayer material was obtained by sequential electrospinning of HA-PEO onto a freshly formed CS-PEO layer; the ratio of the thickness of the CS:HA layers was 2:1. The resulting thickness of the electrospun materials was between 30 and 50 μm.

### 2.4. Film Preparation

The following solutions were cast to compare the structures of the nonwovens and films: CS, CS–PEO, HA, HA–PEO, and a CS–HA bilayer film. The films were formed on a balanced glass substrate by casting from a spinneret. The bilayer composite was formed by the sequential deposition of a solution of HA onto the CS gel film.

### 2.5. General Methods

The swelling of the nonwoven material in water and in a physiological solution was determined by the gravimetric method.

Scanning electron microscopy (SEM) was carried out on a Phenom G Pro (Phenom-World BV, Eindhoven, the Netherlands).

Atomic force microscopy (AFM) was performed using a Smena (NT-MDT, Zelenograd, Russia). Samples were scanned in semi-contact mode with a curvature tip radius of 10 nm, a probe resonant frequency of 190 kHz, and a force constant of 58 N/m.

X-ray diffraction analysis was conducted with a DRON-3M (Burevestnik, St. Petersburg, Russia) using Ni-filtered Cu Kα radiation (λ = 1.5418 Å).

The porosity was determined by standard porosimetry using a Porotech 3.1 instrument (Porotech Ltd., Vaughan, ON, Canada). The experiment involved measurement of the equilibrium curve of the relative moisture content *ν* (the ratio between the volume of liquid in pores and the mass or volume of a porous object) between a standard and the studied sample. In other words, we determined the equilibrium dependence of a relative amount of measuring liquid in the studied sample on its amount in a standard, which had a known porosimetry curve. The standards and the sample were previously dried, weighed, and impregnated with the measuring liquid (octane) under vacuum. After the free liquid was removed, the porous bodies were brought into contact, and a certain amount of the measuring liquid was removed from this set of porous bodies. After the capillary equilibrium was set between the porous bodies, the standards and the sample were weighed separately. The mass and volume of the liquid contained in the standards and the studied sample were determined by comparing their masses with those of dry standards and samples. These steps were conducted until the porous sample was free of liquid. The obtained data were used to plot the required dependence of the relative moisture content in the studied sample on the moisture content in the standard. This dependence and the calibration porosimetry curves for the standards (distribution of pore radii) were used to obtain a porosimetry curve for the studied sample. This basic curve was processed using the software Porovoz, which provides a vast amount of information about the porous structure of a sample.

The mechanical tests were carried out in the uniaxial extension mode at room temperature (20 °C) using an AG-100kNX Plus universal mechanical test system (Shimadzu, Kyoto, Japan). Strip-like samples 20 × 2 mm in size were stretched at a rate of 10 mm/min, according to the ASTM D638 requirements.

### 2.6. Cell Culture and in Vitro Tests

All in vitro biological tests were performed according to the Declaration of Helsinki, and approval was obtained from the Ethics Committee of the Almazov National Medical Research Centre (no. 12.26/2014; December 1, 2014). Written informed consent was obtained from all subjects before fat tissue biopsy. Fat-derived multipotent MSCs from healthy donors had the following phenotype: CD19-, CD34-, CD45-, CD73+, CD90+, CD105+, as confirmed by flow cytometry (GuavaEasyCyte8; MerckMillipore, Darmstadt, Germany) and monoclonal antibodies (Becton Dickinson, Franklin Lakes, NJ, USA). The cells were maintained at 37 °C and 5% CO_2_ in α-MEM medium (PanEco, Moscow, Russia) supplemented with 10% fetal calf serum (HyClone Laboratories, Inc., Logan, UT, USA), 50 units/mL penicillin, and 50 μg/mL streptomycin (Invitrogen, Waltham, MA, USA). 

The biocompatibility of the materials (cell adhesion and viability) was determined as previously described [43] using MSCs from passages 4–5. Material samples measuring 12 × 8 mm were co-cultivated with MSCs in the wells of a 24-well plate for three and six days. Cells were seeded at a density of 5 × 10^4^ cells per well. In the case of the CS–HA bilayer scaffold, cells were seeded onto HA surface. Cover glasses were used as the control. Cell adhesion and attachment were analyzed by studying intracellular vinculin expression using fluorescence microscopy. Samples with cells after incubation were removed from the medium, and the cells were washed with phosphate buffer saline (PBS) and fixed with 4% paraformaldehyde for 20 min. These cells were permeabilized using Triton X-100, rinsed with PBS, blocked with 10% goat serum in PBS for 30 min at room temperature, and incubated with an anti-vinculin antibody (Thermo Fisher Scientific, Waltham, MA, USA) at 1:200 dilution for one hour. After three PBS washes, the cells were incubated with Alexa Fluor 568 goat anti-mouse IgG (H + L) (Invitrogen, Waltham, MA, USA) at 1:1000 dilution for one hour at room temperature in the dark. The cells were washed thrice with PBS (5 min each) and stained with 4′,6-diamidino-2-phenylindole (DAPI) for visualization of the nuclei. The cells were washed and then viewed using an AxioObserver fluorescence microscope (Carl Zeiss, Jena, Germany). Images were collected and processed with the software ZEN. Samples were visualized using ×40 magnification to investigate cell morphology and adhesion processes. The quantitative analysis of adhered cells was investigated at a magnification of ×10. The number of nuclei (DAPI positive) was calculated in at least 10 fields of view and then recalculated as number of cells per 1 mm^2^. Cell viability was verified after the co-culturing with the material samples as follows. The cells were removed from the material using trypsin, resuspended in an annexin binding buffer (BioLegend, San Diego, CA, USA), and stained with annexin V-FITC (BioLegend, San Diego, CA, USA) and propidium iodide (Sigma-Aldrich, St. Louise, MO, USA) for 20 min in the dark. Flow cytometry was performed using the GuavaEasyCyte8 (MerckMillipore, Darmstadt, Germany) instrument by determining the relative percentages of double-positive (late apoptosis/necrosis), double-negative (living), and annexin V-positive (early apoptosis) cells. 

All biological tests were run at least in triplicate. Statistical analysis was performed using the software STATISTICA 7.0 (StatSoft, Tulsa, OK, USA) and GraphPad Prism (GraphPad Software Inc., San Diego, CA, USA). Data are presented as mean ± standard error. Statistically significant differences were calculated using the Mann–Whitney U-test; a value of p <0.05 was considered statistically significant.

## 3. Results

### 3.1. Preparation and SEM Morphology of CS and CS–HA Nonwoven Materials

**Figure 1:**
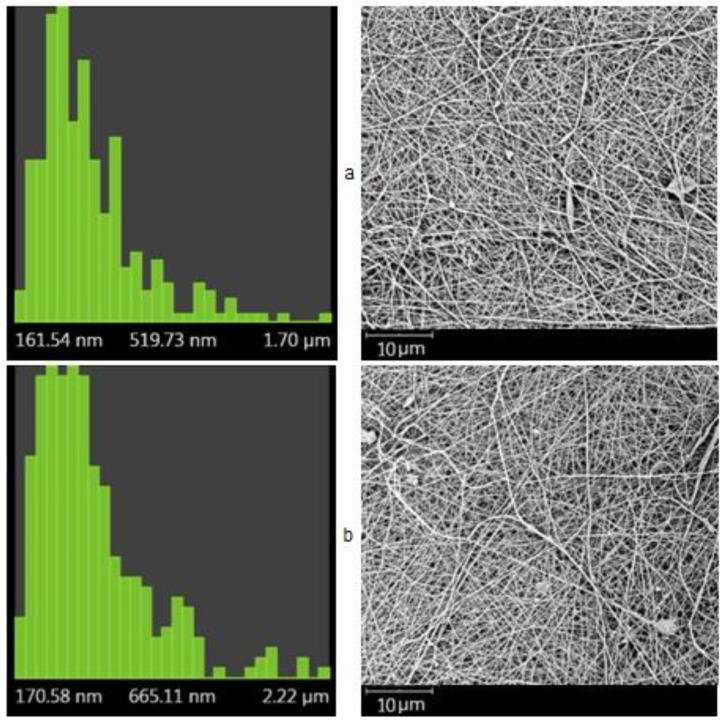
Distribution of the fiber diameter and the fiber morphology of the chitosan (CS) surface (**a**) and the hyaluronic acid (HA) surface (**b**) of the bilayer nonwoven material. Distribution parameters were calculated from three SEM images (a total of 250–300 measurements).

The electrospinning parameters described in the Materials and Methods section (voltage of 60–75 kV, distance between electrodes of 22–24 cm, and rotation speed of spinning electrode of 10–16 min^−1^) were experimentally selected for each solution to ensure spinning stability and material uniformity. The solution composition and concentration were optimized to produce fibers in the diameter range of about 200–800 nm, as suggested in the previous studies [44,45,46]. Figure 1 shows the distribution of the fiber diameters for the bilayer nonwoven CS–HA composite; the average diameters of the fibers ranged from 360 to 420 nm for the CS side and from 370 to 650 nm for the HA side. The SEM images of the CS–HA bilayer composite had a reasonably uniform fiber formation and did not have beads.

### 3.2. Swelling

The obtained bilayer nonwoven material contained a layer of CS and a layer of HA, as well as a water-insoluble PEC that formed during contact of oppositely charged nanofibers in the process of multilayer spinning. The hydrophilicity of the obtained bilayer nonwoven material (Table 1) was determined by the properties of the initial polymers and the changes they underwent during electrospinning and subsequent processing. The intense intermolecular interactions occurring between CS and PEO during electrospinning caused a partial loss of solubility in water in the nonwoven material based on CS [47,48]. Subsequent heating of the nonwoven material led to the loss of solubility and a reduction in the degree of swelling in water and the physiological solution (Table 1, entry 1); these changes were associated with the formation of amide crosslinks [49].

The partial loss of solubility by the CS–HA bilayer composite immediately after electrospinning may be due to the formation of interpolymeric complexes of polysaccharides with PEO [35] or by the formation of PEC between the cationic CS and the anionic HA. The CS–HA PEC initiated a polymorphic modification of anhydrous CS, thereby reducing the hydrophilicity of the multilayer composite [38]. Heating decreased the degree of swelling of the nonwoven bilayer CS–HA in water and in the physiological solution (Table 1, entries 2 and 3), and this finding may be associated with an acceleration of structural changes. These bilayer samples retained their shape, despite the high degree of swelling in water and reduced swelling in the physiological solution. This phenomenon could be indicative of a useful property for cell culture scaffolds in therapy, where desired implant shapes must be maintained [50]. 

### 3.3. X-Ray Diffraction

**Figure 2:**
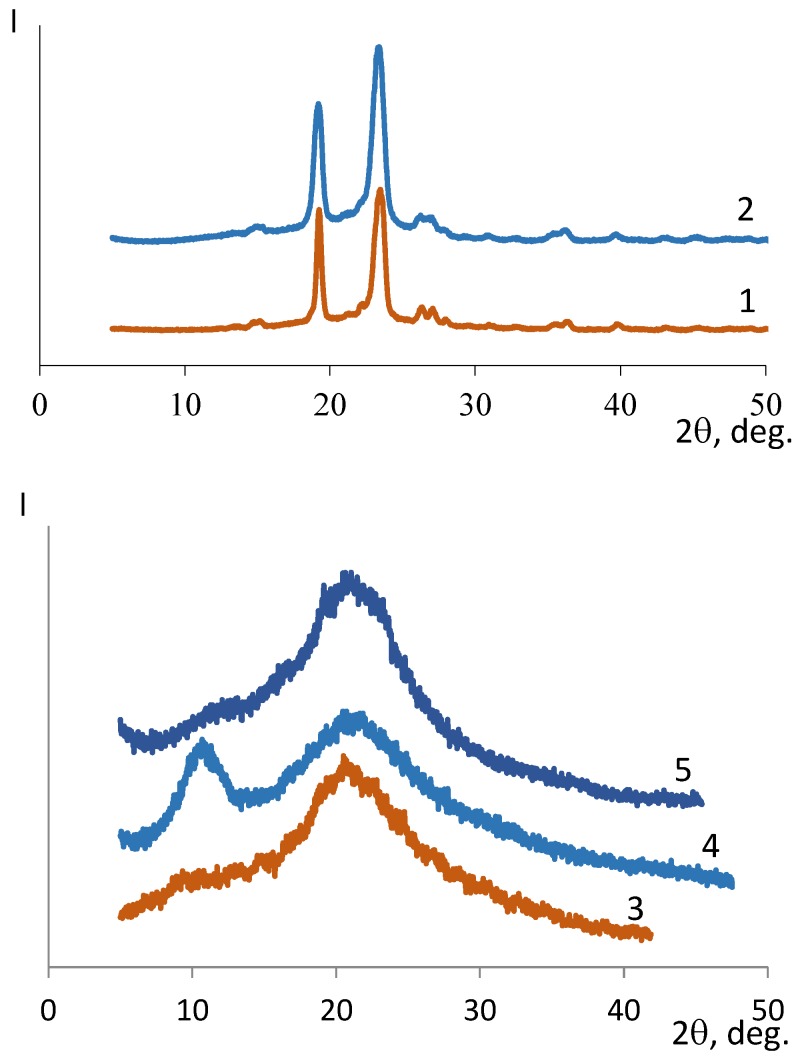

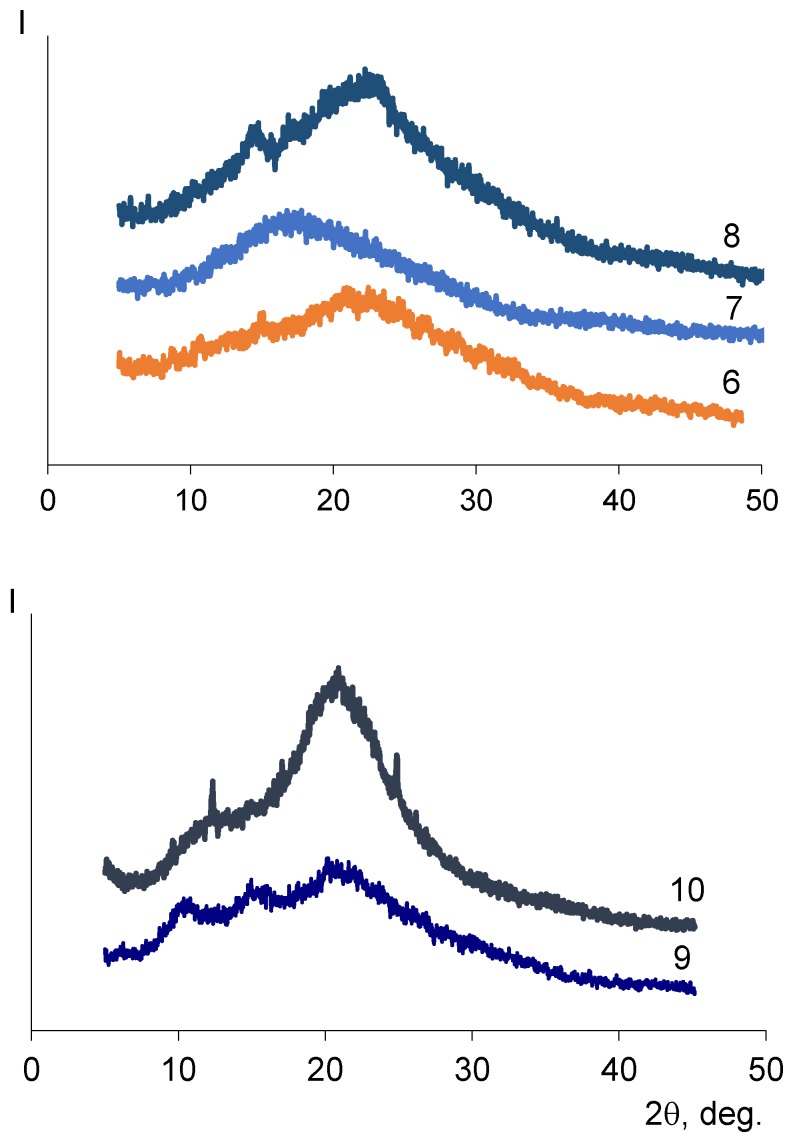
X-ray diffraction patterns: polyethylene oxide (PEO)-film (1), PEO-nonwoven (2), CS-film (3), CS–PEO‑film (4), CS–PEO-nonwoven (5), HA-film (6), HA–PEO-film (7), HA–PEO-nonwoven (8), CS–HA‑film (9), CS–HA–PEO-nonwoven (10).

The effect of electrospinning on the polymer structure was examined by conducting a comparative X-ray diffraction analysis of the initial polymers and the bilayer composites in the form of films and nonwoven materials. Effective polysaccharide electrospinning requires PEO; therefore, X-ray diffraction was used to analyze the PEO films and nonwoven materials. These samples demonstrated high degrees of crystallinity of 96% and 85%, respectively (Figure 2).

The structures of the initial polymers in the form of films with and without PEO were compared (Figure 2). The introduction of PEO into the CS film (Figure 2) led to the appearance of a signal at 2θ = 10° (the reflexion of the hydrated CS) and a slight signal broadening at 2θ = 22°. For the HA films, the reflexion of HA at 2θ = 23° with the introduction of PEO was shifted to the 2θ = 18° region, which is specific for PEO (Figure 2). The reflexions were less pronounced compared with those for the mixture of HA and PEO polymers; this finding may indicate an interaction between PEO and the polyacid. 

A comparison of the structures of the CS–PEO composite film and nonwoven material (Figure 2) revealed an amplification of the signal intensity at 2θ = 22° and disappearance of the signal at 2θ = 10°, characterizing the hydrated CS polymorph. This may indicate the occurrence of strong intermolecular interactions during electrospinning, leading to changes in hydrophilicity.

Unlike a bilayer film, the electrospun bilayer matrix showed a noticeably increased intensity of the signal at 2θ = 22–23° (specific for both CS and HA) and the emergence of a weak signal (a shoulder) at 2θ = 15° (Figure 2). This latter signal is specific for the anhydrous CS polymorph [51], as the appearance of anhydrous CS is initiated by the formation of a PEC between CS and HA layers [38]. This signal has a lower intensity than that in a bilayer film because a PEC is formed only at the sites of local contacts of the polyelectrolytes in nanofibers.

The change in the structure of the nonwoven material can be assumed to be associated with the orientation of the macromolecules of the polymers during electrospinning, which results in strong intermolecular interactions. Meanwhile, the interactions of the polymers with PEO (a highly crystalline polymer capable of chemical interactions with both CS and HA) are enhanced. Intramolecular crosslinking is also possible for HA due to its structure and the interaction of the carboxyl groups and the weakly basic hydroxyl groups of HA. 

### 3.4. Porosity

Porosity is a vital scaffold parameter that influences cellular responses [52]. For example, Hofmann et al. [53] demonstrated that pore size distribution influences cell adhesion and increases intracellular interactions mainly due to the connections between pores. They found that the pore structure determines where the cells are seeded. Moreover, the structure of tissue-engineered bone is controlled by the underlying scaffold geometry, which affects the in vivo cell viability and implant compatibility. Pore connections are essential for tissue vascularization and for nutrient supply and metabolite diffusion, which promote the viability and growth of the cells situated deep within the material [54,55]. 

The electrospun monolayer CS and bilayer CS–HA matrices had a large inner surface (Table 2) and a well-developed pore structure, which can benefit cell adhesion, migration, proliferation, and differentiation. Improvements in these cell responses would be advantageous for tissue engineering. The CS and CS–HA matrices had similar average pore radii. The monolayer CS matrix had a significantly higher total porosity compared with the bilayer CS–HA, and the total pore surface decreased dramatically with the addition of the HA layer (Table 2), suggesting the formation of close contact between HA and CS in the form of a PEC. Moreover, the electrospun scaffolds had significantly higher porosity compared with the CS film (Table 2; [32]), and this would allow the formation of multiple junctions with the cell.

### 3.5. Mechanical Tests

The mechanical characteristics of the tested samples, along with their thickness d, are listed in Table 3. The values of Young’s modulus E, yield stress σ_y_, break stress σ_b_, and ultimate deformation ε_b_ were calculated using the parameter of the sample cross-section, without taking into account the porosity of the materials.

**Figure 3:**
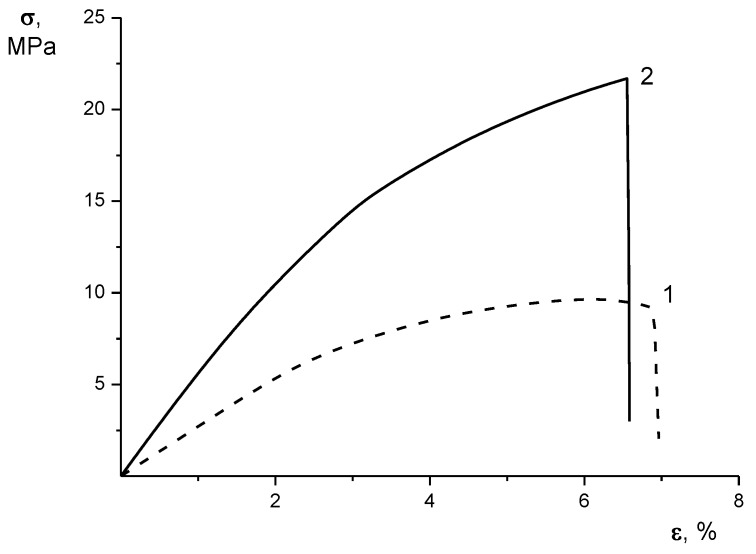
Stress-strain curves of the CS (1) and CS–HA (2) nonwoven materials.

Both the E and σ_b_ values of the bilayer CS–HA nonwoven material substantially exceed (by more than two fold) the values of the CS nonwoven. The ultimate deformation ε_b_ of CS was only 0.4% higher than this characteristic of CS–HA. This difference could be disregarded, but the rupture of both materials took place in precisely the same deformation range as that of the realization of the yield process (σ_y_). As clearly demonstrated by the stress-strain curves (Figure 3), the breaking process in the CS nonwoven took place in the deformation range beyond the realization of the yield point, whereas CS–HA broke before this event. Thus, the bilayer CS–HA nonwoven material, with its reinforcing PEC layer, shows increased strength and stiffness when compared to monolayer CS nonwoven material.

### 3.6. Roughness

The roughness of the scaffold is an essential parameter that determines effective cell proliferation. However, comprehensive analysis has been performed for relatively stiff surfaces (such as hydroxyapatite [56], polystyrene [57], or titanium [58]) with bone cell cultures only, and the findings generally revealed an increase in cell proliferation along with surface roughness. The surface roughness of hydroxyapatite improved the short- and long-term responses of human bone marrow cells. Cell adhesion, proliferation, and detachment strength were sensitive to the surface roughness and increased as the roughness of hydroxyapatite increased from 0.73 μm to 4.68 μm [56]. For polystyrene, the surface roughness caused increases in osteoblastic proliferation and differentiation in cell cultures. The proliferation and gene expression of alkaline phosphatase (ALP) and osteocalcin in immature osteogenic rat cells increased when the cells were placed on rough-surfaced cover strips with an average roughness of up to 0.8 μm; the cellular responses then decreased to the level observed for a smooth surface [57]. By contrast, another study found that a high roughness of 0.6–6 μm on titanium substrates did not affect cell proliferation and differentiation, according to an evaluation of the DNA and alkaline phosphatase synthesis in rat bone marrow cells [58].

**Figure 4:**
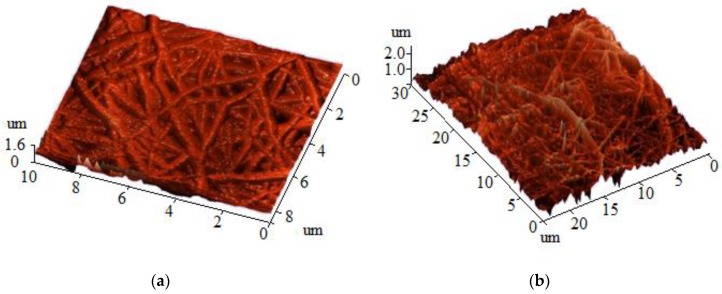
AFM imaging of the surface of the nonwoven material’s (**a**) HA-face and (**b**) CS-face.

The average roughness of the electrospun CS–HA mats was determined by AFM (Figure 4). The roughness of the bilayer CS–HA material was about 1.5–3 μm, which is within the optimum range for effective cell growth, according to previous studies [56,57,58]. No significant differences were observed in the roughness of the CS-face and the HA-face. 

### 3.7. Biocompatibility Testing

**Figure 5:**
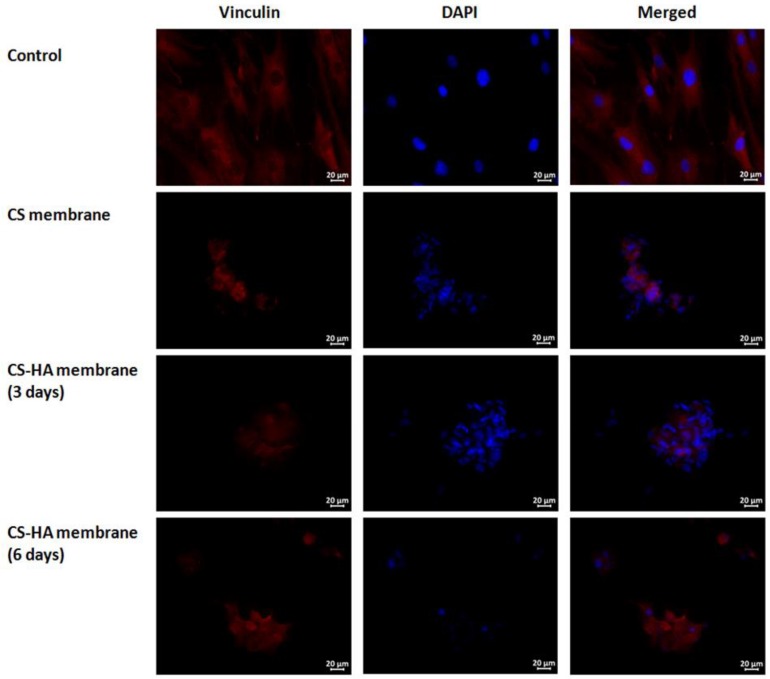
Representative cell morphology after three days of co-cultivation of mesenchymal stem cells (MSC) and cover glass (first row), CS scaffold (second row), and CS–HA scaffold (third row) and after six days of co-cultivation with CS–HA scaffold (fourth row).

After three days of co-cultivation, the numbers of the cells and their shapes and adhesion types on both scaffolds were identical; the cells were located on the surface, alone or in small spheroid-like colonies (Figure 5). The cells had a typical round shape, but a few were branched or spindle cells. Essentially none of the cells spread on the surface of the material showed a typical stellar shape. Staining for vinculin, an intracellular adhesion protein, revealed a diffuse distribution over the cytoplasm. No focal adhesion junctions were visualized. The CS–HA scaffold surface had more cells than the CS scaffold surface, but the difference was not statistically significant.

After six days of co-cultivation, the number of cells on the CS–HA scaffolds was the same as that after three days, but their morphology differed. Cells were also located on the surface, alone or predominantly as spheroid-like colonies. The spheroids were large with the increased number of cells. Meanwhile, cells on the periphery of the spheroids that were spread on the material were bigger and had an increased number of branches (Figure 5). 

**Figure 6:**
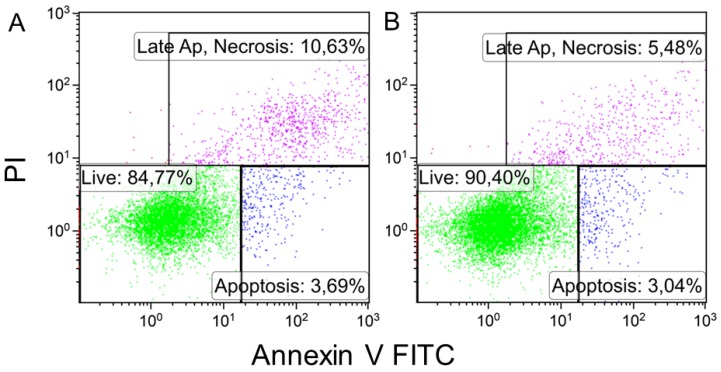
Biocompatibility testing results after three days of co-cultivation of MSCs and scaffolds. Representative dot plots of cell samples stained with Annexin V FITC and propidium iodide. (**A**) Cells co-cultivated with CS scaffolds. (**B**) Cells co-cultivated with CS–HA scaffolds.

An assessment of the viability of the cells that adhered onto the surface of the material demonstrated a significantly higher number of living cells on the surface of the CS–HA scaffold compared with CS scaffold (*p* = 0.05) and a considerably reduced number of cells in the irreversible stage of late apoptosis or necrosis (Table 4, Figure 6). According to this observation, together with the adhesion of the MSCs onto the surface of the material, a logical conclusion is that the CS–HA scaffold had slightly better biocompatible properties compared with the monolayer CS scaffold.

## 4. Discussion

The adhesion of cells onto the surface of a material is a complex multistage process that depends mostly on the nature of the surface itself, including its chemical properties and topographical features [19,43]. The adhesion can proceed in three possible scenarios. In the first case, the cells interact with the surface but do not adhere. In the second case, passive adhesion occurs, in which cells can interact and adhere but are easily detached by minor damage or exposure [59,60]. The pattern of the adhesion largely depends on the physicochemical interactions between the surface of the material, the adsorbed proteins, and the adherent cells. This kind of adhesion is reversible. The third option is characterized by active adhesive interaction, in which cells firmly adhere to the surface of the material. The interaction between the cell membrane receptors and the surface of the material leads to the transformation of the cell shape; it sprawls and flattens [61]. The sprawled cells can spontaneously detach from the surface, but this does not lead to any crucial changes in the cell microenvironment [19].

According to the type of adhesive process, two types of surfaces can be distinguished: inert and adhesive. For the adhesive type, the cells are attached due to both passive and active adhesion. In the first stage, the cells adhere to the substrate due to physicochemical interactions, including hydrophobic, Coulomb, and van der Waals forces [62]. The adhesion interactions in this phase are passive. Active adhesion then occurs through the binding of the cell integrin, and the cell begins to sprawl and flatten. In the third phase, the cytoskeleton is organized with the formation of focal adhesion junctions between the cell and the surface of the substrate. 

During cell migration and in the initial stage of the formation of contact with the substrate surface, intracellular vinculin is localized pointwise at the sites of cell attachment to the substrate (focal contacts). The focal adhesion sites elongate, and fluorescent labeling with vinculin antibodies shows that they take the form of short bands [63]. Thus, intracellular vinculin is not visualized in the form of diffuse staining; instead, it appears as short bright bands, and this feature serves as a reliable marker of the active adhesive process and active cell–material interactions. In the present study, a sufficiently large number of adhered cells, some of which tended to spread, indicated that both the CS and CS–HA matrices were adhesive. However, at the same time, the small number of spread cells and the absence of focal adhesion junctions after co-cultivation for three days indicated that most of the cells were still in the process of turning from passive to active adhesion. Meanwhile, the change in MSC morphology that occurred after six days with CS–HA co-cultivation demonstrated that adhesion was active but stretched in time.

The assembly of MSC spheroids on CS membranes has been reported [64]. In this case, the process differs from the adhesion that occurs in suspension or on a non-adherent polymer surface. MSCs are first attached to CS membranes, and they spread through the membranes. The pseudopodia are then drawn up, and the cells form multicellular spheroids [65,66]. 

One important feature to note is that the ability to differentiate increases in stem and progenitor cells under 3D culture conditions, such as spheroids. For example, progenitor cells derived from the salivary glands can differentiate into hepatocytes and cells of pancreatic islets, but this is possible only when cells are cultured in aggregates of 3D cells and not in 2D monolayers [67]. Spheroids formed from bone marrow MSCs have enhanced anti-inflammatory properties [68]. 

Cultivation of MSCs on the surfaces of materials in the form of spheroids allows for control of differentiation. For example, the formation of spheroids on the surface of CS and CS–HA films can maintain the expression of stem marker genes [65]. Bone marrow MSC spheroids have demonstrated highly efficient osteogenic [69] and adipogenic differentiation [69,70]. Aggregation of a dense mass of cells creates an environment with strong intercellular interactions, which promotes the immediate differentiation of MSCs into chondroblasts [71]. 

HA is used in tissue engineering to create scaffolds that can control chondrogenesis [72] and provide a favorable niche for stem cell chondrogenesis in vitro and in vivo [73,74]. At the same time, the cultivation of MSCs on CS and CS–HA matrices and the resulting induction of chondrogenic differentiation lead to an increased expression of the *SOX9*, *aggR*, and collagen II (*COL2A1*) genes. Thus, the role of CS and CS–HA matrices can differ; a microenvironment is created either to support MSC potential or to stimulate chondrogenesis. 

## 5. Conclusions

A bilayer nonwoven material based on CS and HA was obtained by electrospinning. The material kept its shape while swelling in water due to intramolecular interactions during electrospinning and the formation of a net of local contacts between the CS and HA layers. The bilayer CS–HA matrix had a lower porosity (59% v/v) and a smaller mean pore size (416 nm) than a monolayer CS matrix (98% v/v and 489 nm, respectively). The surface roughness of the matrices was 1.5–3 μm. Biocompatibility tests with MSCs revealed the formation of MSC spheroids on the surface of the matrices. The viability of the MSCs and the observed cell adhesion on the surface of the materials led to the conclusion that the CS–HA matrix had slightly better biocompatibility than did the CS matrix. Therefore, the scaffolds fabricated in the present study have high therapeutic potential as tissue engineering matrices.

## Figures and Tables

**Table 1:** Swelling of nonwoven materials, mean ± standard deviation (n = 3).

Entry	Sample	Treatment	Swelling in Water, g/g	Swelling in 0.9% NaCl, g/g
1	CS-nonwoven	80 °С, four hours	5.3 ± 0.4	2.2 ± 0.2
2	CS–HA-nonwoven	80 °С, four hours	1.4 ± 0.1	1.0 ± 0.1
3	CS–HA-nonwoven	100 °С, two hours	2.8 ± 0.2	2.3 ± 0.2

**Table 2:** Porosity of materials.

Parameter	CS-Film [32]	CS-Nonwoven	CS–HA-Nonwoven
Average logarithmic pore radius, nm	1.17	1.62	1.89
Average pore radius, nm	241	489	416
Porosity over weight, cm^3^/g	0.29	9.36	6.02
Porosity over volume, cm^3^/cm^3^	0.29	0.976	0.587
Meso- and macro-pore surface over weight, m^2^/g	29.9	878	950
Meso- and macro-pore surface over volume, m^2^/cm^3^	29.2	89.1	92.7
Total pore surface over weight, m^2^/g	23.3	2464	1027
Total pore surface over volume, m^2^/cm^3^	22.8	257	100

**Table 3:** Mechanical properties of nonwoven materials, mean ± standard deviation (n = 3).

Sample	d, μm	E, MPa	σ_y_, MPa	σ_b_, MPa	ε_b_, %
CS-nonwoven	40–42	253 ± 23	9.7 ± 0.7	9.4 ± 0.8	6.9 ± 0.8
CS–HA-nonwoven	30–31	555 ± 25	-	22.2 ± 0.9	6.5 ± 0.3

*d—sample thickness, E—Young’s modulus, σ_y_—yield stress, σ_b_—break stress, and ε_b_—ultimate deformation.

**Table 4:** Biocompatibility testing results after three days of MSC and scaffold co-cultivation, mean ± standard error (n ≥ 3).

Sample	Adhered Cells, cells/mm^2^	Living Cells, %	Late Apoptosis/Necrosis, %	Early Apoptosis, %
CS-nonwoven	281 ± 18	84.1 ± 1.8^1^	10.2 ± 1.2^1^	4.7 ± 0.5
CS–НА-nonwoven	397 ± 39	89.6 ± 0.8^1^	5.3 ± 0.3^1^	3.6 ± 0.5

^1^*p* = 0.05.
